# Supplementary material for: Arsenic binds to nuclear transport factors and disrupts nucleocytoplasmic transport
Source: J Cell Sci. 2025 Aug 15;138(16):jcs263889. doi: 10.1242/jcs.263889 (PMC12401540; doi:10.1242/jcs.263889)
Supplement: Supplementary information [file joces-138-263889-s1.pdf]

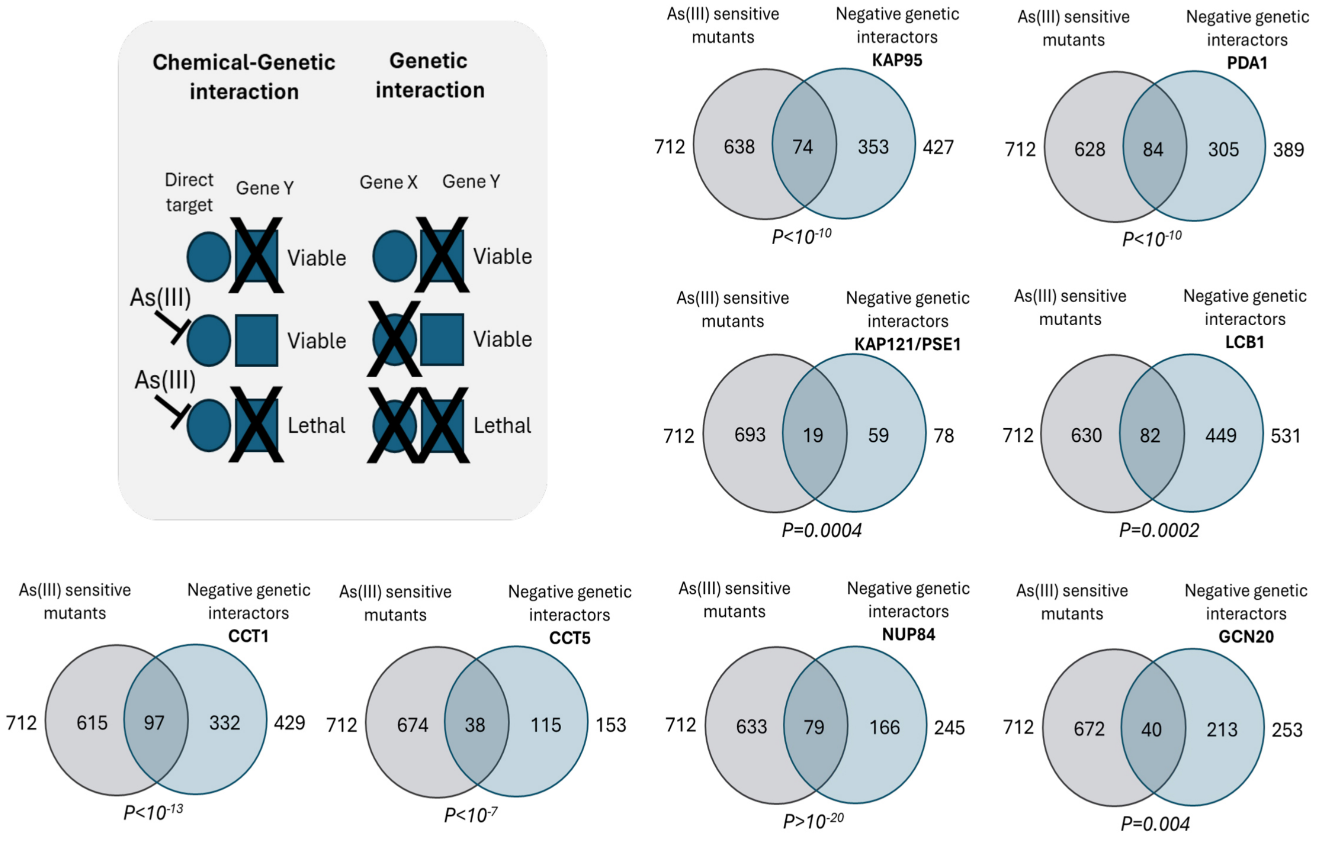

Fig. S1. Integration of chemical-genetic and genetic interaction data to identify *bona fide* arsenic toxicity targets. Negative genetic interactors (including negative genetic, synthetic growth defect, synthetic lethality) of selected arsenic-binding hits were retrieved from SGD (Wong et al., 2023) and compared to a compendium of 712 As(III) sensitive *S. cerevisiae* mutants that contains the genes identified at least once in four genome-wide phenotypic screens (Haugen et al., 2004; Pan et al., 2010; Thorsen et al., 2009; Zhou et al., 2009). The significance of the overlaps between the datasets (negative genetic interactor sets and As(III) sensitive set) was calculated by the hyper-geometric test and the corresponding P-values are indicated.

Journal of Cell Science • Supplementary information

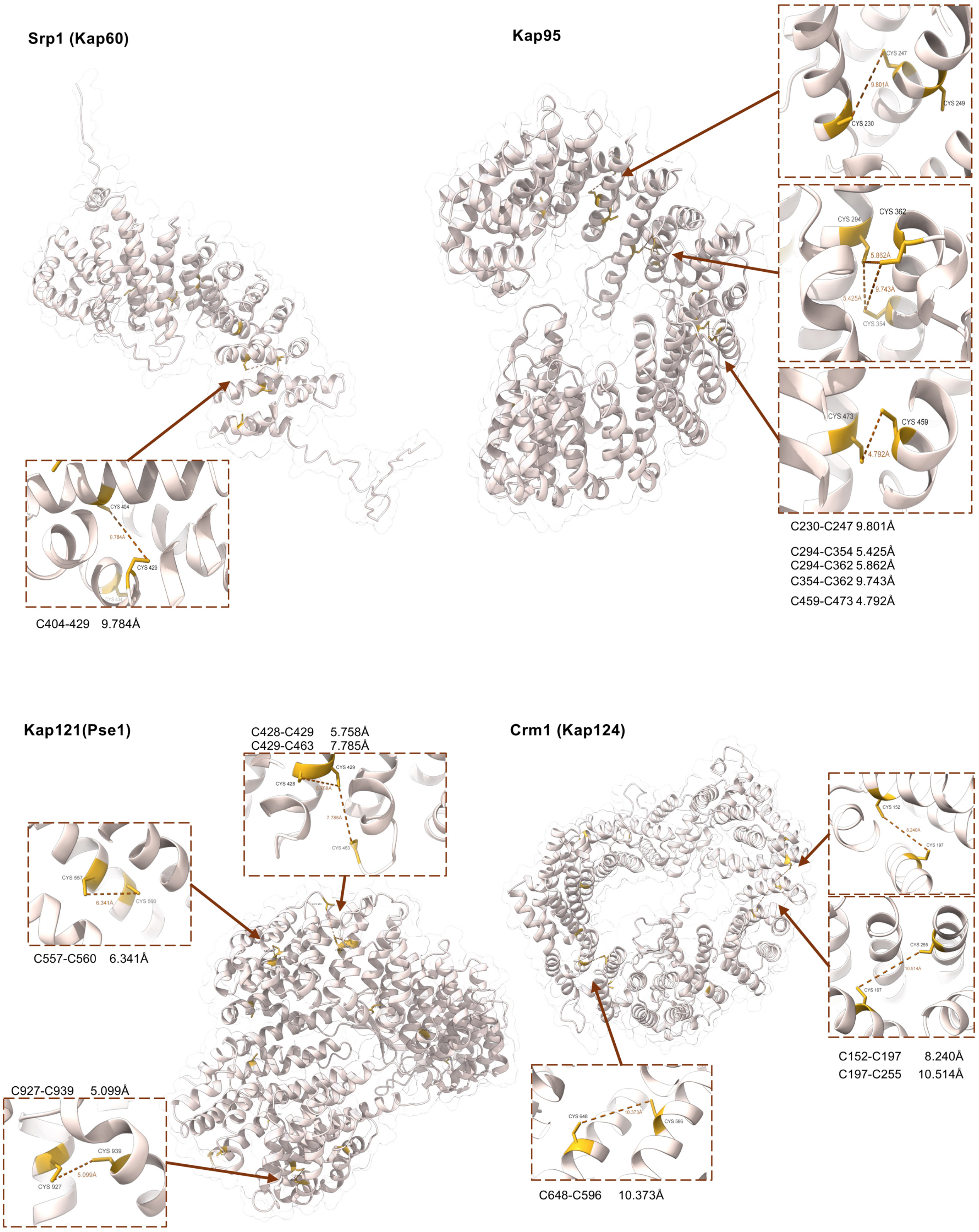

Journal of Cell Science • Supplementary information

Kap123

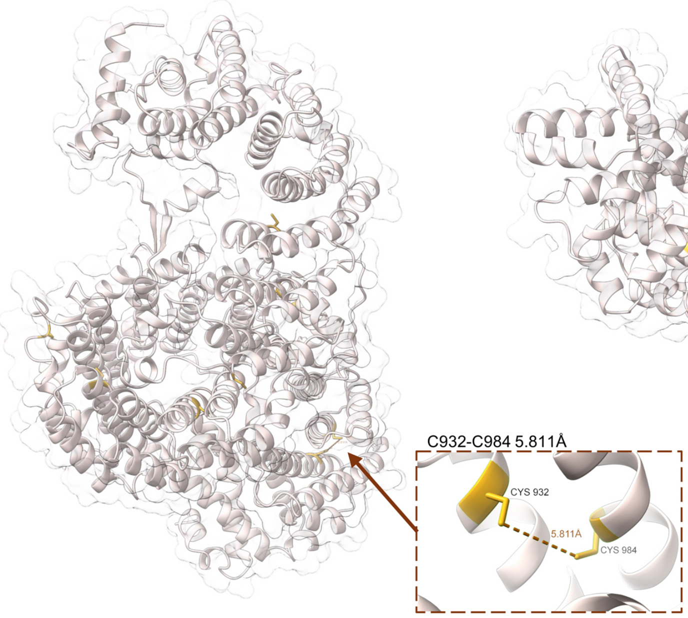

Kap108(Sxm1)

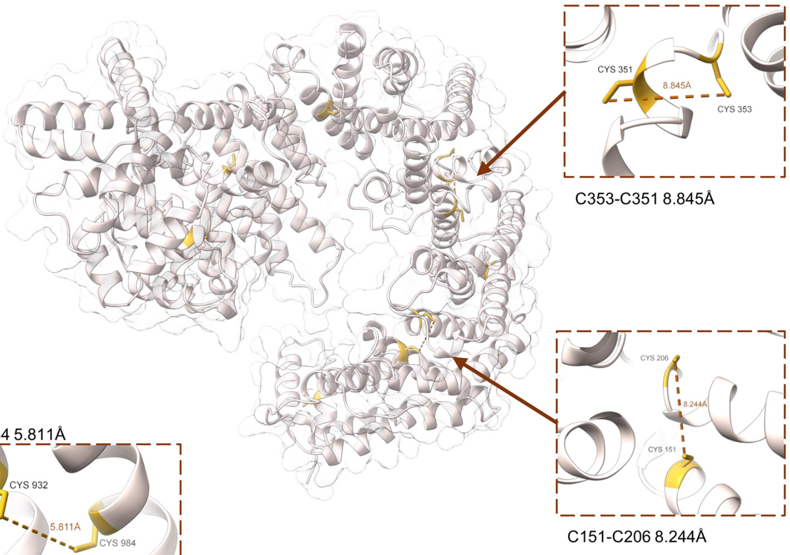

Msn5

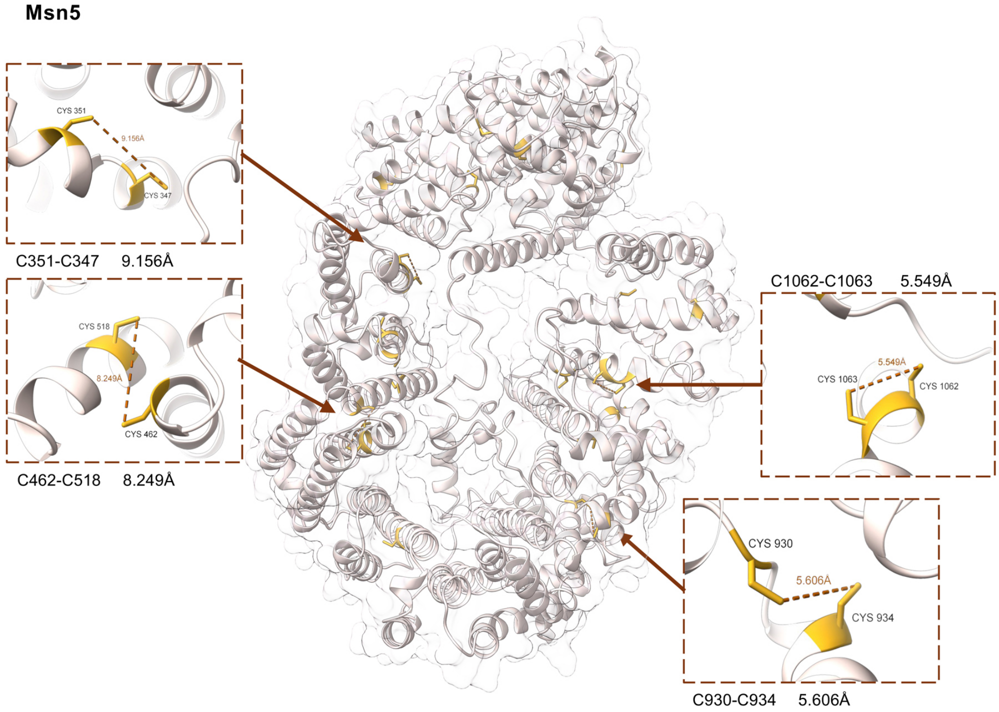

Journal of Cell Science • Supplementary information

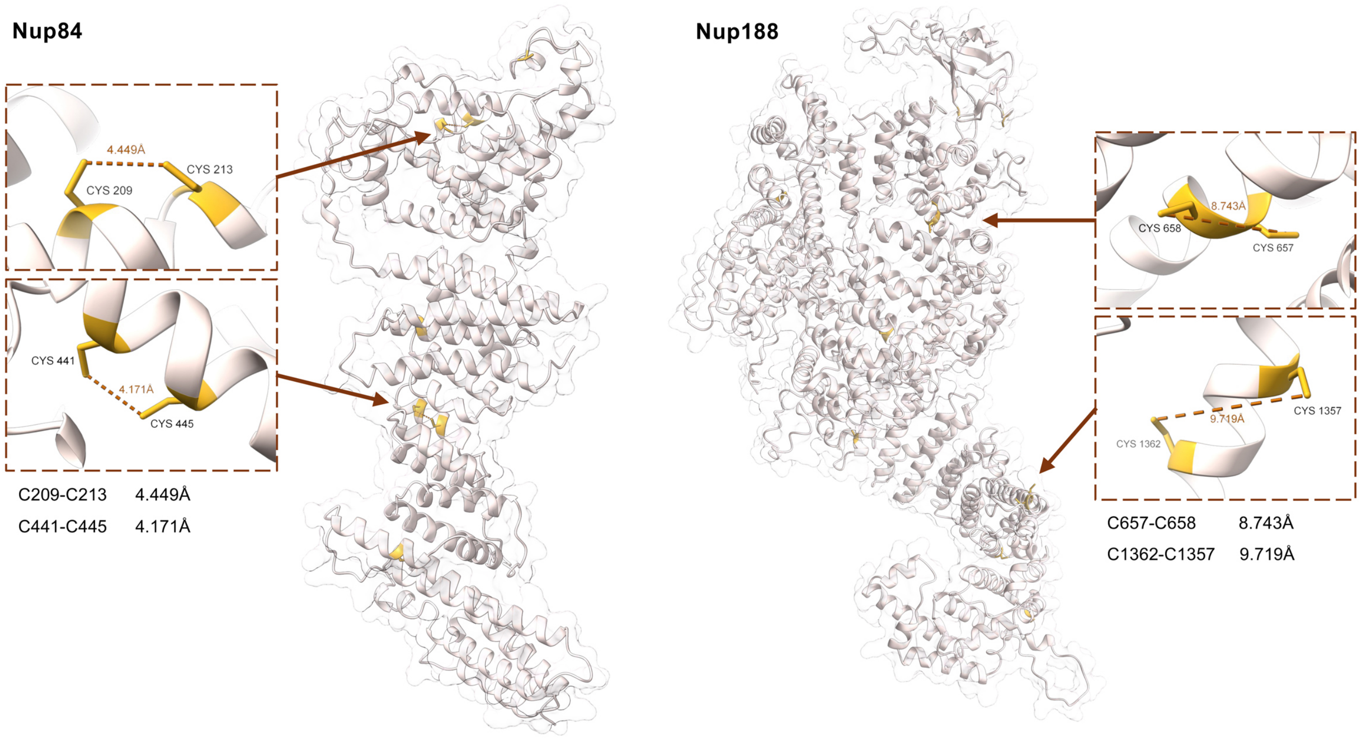

Fig. S2. AlphaFold structure predictions and cysteine mapping for yeast Srp1, Kap95, Kap121/Pse1, Crm1, Kap123, Sxm1/Kap108, Msn5, Nup84 and Nup188. Distances between pairs of adjacent or proximal cysteines are indicated. The structure predictions are based on experimental crystal structure data for all proteins except for Kap123, Kap108/Sxm1 and Msn5.

Journal of Cell Science • Supplementary information

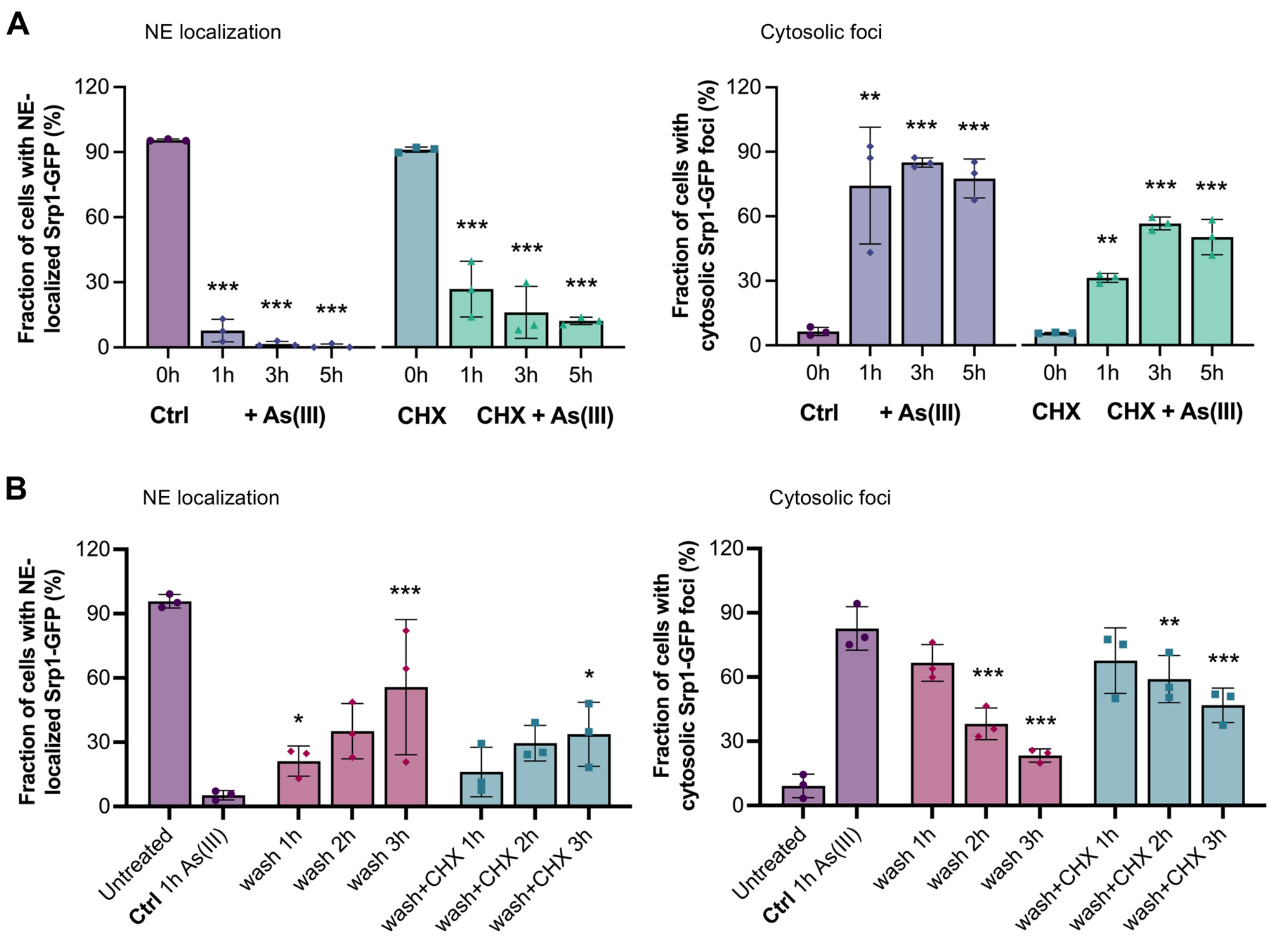

Fig. S3. Mislocalization of Srp1 in As(III)-exposed cells. (A) Quantification of Srp1-GFP nuclear envelope (NE) localization (left panel) and foci formation (right panel) in the absence and presence of 1.5 mM As(III) for 1 h and/or 0.2 mg/ml cycloheximide (CHX). Srp1–GFP distribution was scored by fluorescence microscopy and quantified by visual inspection. The bars represent the mean ± SD of three independent biological repeats of a total of 300 cells. Significance was calculated using un-paired two-tailed student's t-test with either the untreated control (for just As(III) exposure) or CHX (for CHX+As(III) treated cells) as the comparison, and P-values are according to: ** > 0.01, *** > 0.001. (B) Cells were exposed to 1.5 mM As(III) for 1 h, then washed twice and resuspended in medium without As(III) in the absence or presence of 0.2 mg/ml CHX. Srp1–GFP distribution was scored by fluorescence microscopy and quantified as in S3A. Significance was calculated using un-paired two-tailed student's t-test of three independent biological replicates, with 1 h As(III)-exposed cells as the control sample. P-values according to * > 0.05, ** > 0.01, *** > 0.001.

Journal of Cell Science • Supplementary information

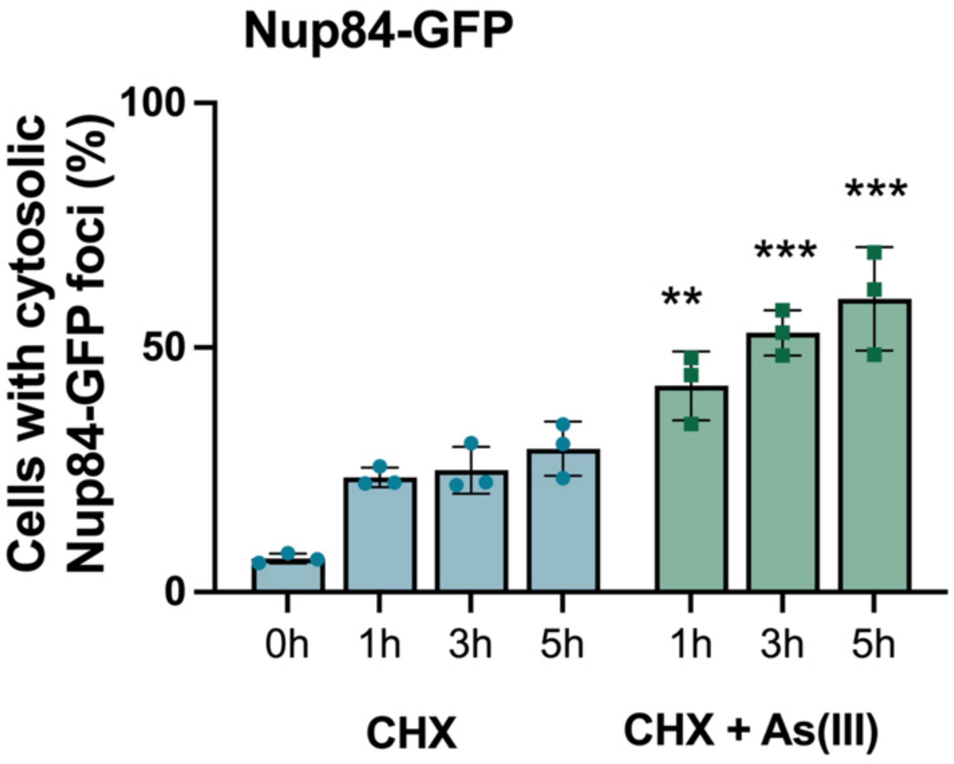

Fig. S4. Cycloheximide does not prevent Nup84-GFP mislocalization in As(III)-stressed cells. Quantification of Nup84-GFP foci formation in the absence and presence of 1.5 mM As(III) for 1 h and/or 0.2 mg/ml CHX. Foci formation was detected with fluorescence microscopy and quantified by visual inspection. The bars represent the mean ± SD of three independent biological repeats of a total of 300 cells. Significance was calculated using un-paired two-tailed student's t-test with CHX-treated cells as the comparison, and P-values are according to: ** > 0.01, *** > 0.001.

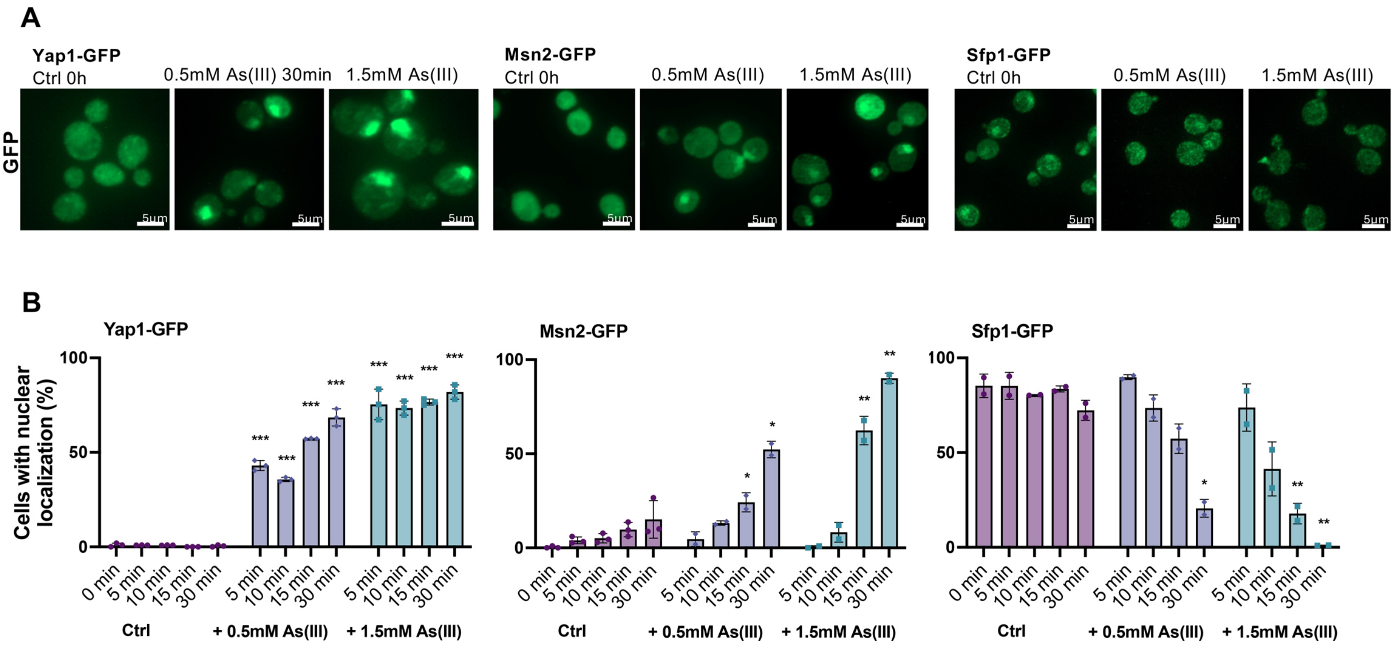

Fig. S5. Nuclear transport is functional during short-term As(III) exposure. (A) Cells expressing GFP-tagged versions of the transcription factors Yap1, Msn2, and Sfp1 were either left untreated (Ctrl) or exposed to the indicated concentrations of As(III), and their localization determined by fluorescence microscopy. (B) Quantification was done by visual inspection, and the bars represent the mean ± SD of three independent biological repeats of a total of 300 cells. Significance was calculated using un-paired two-tailed student's t-test with the untreated control at the respective time point as the comparison, and P-values are according to: *> 0.05, ** > 0.01, *** > 0.001.

Journal of Cell Science • Supplementary information

KpnB1

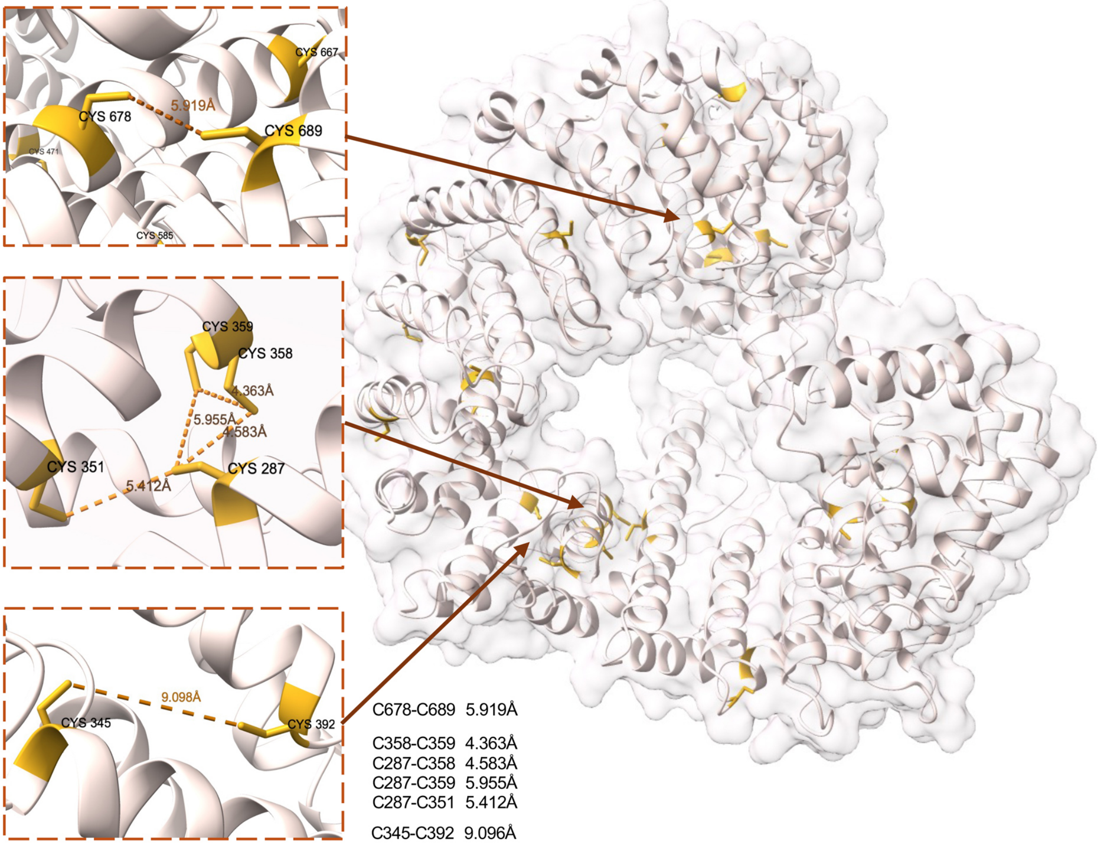

RanB1

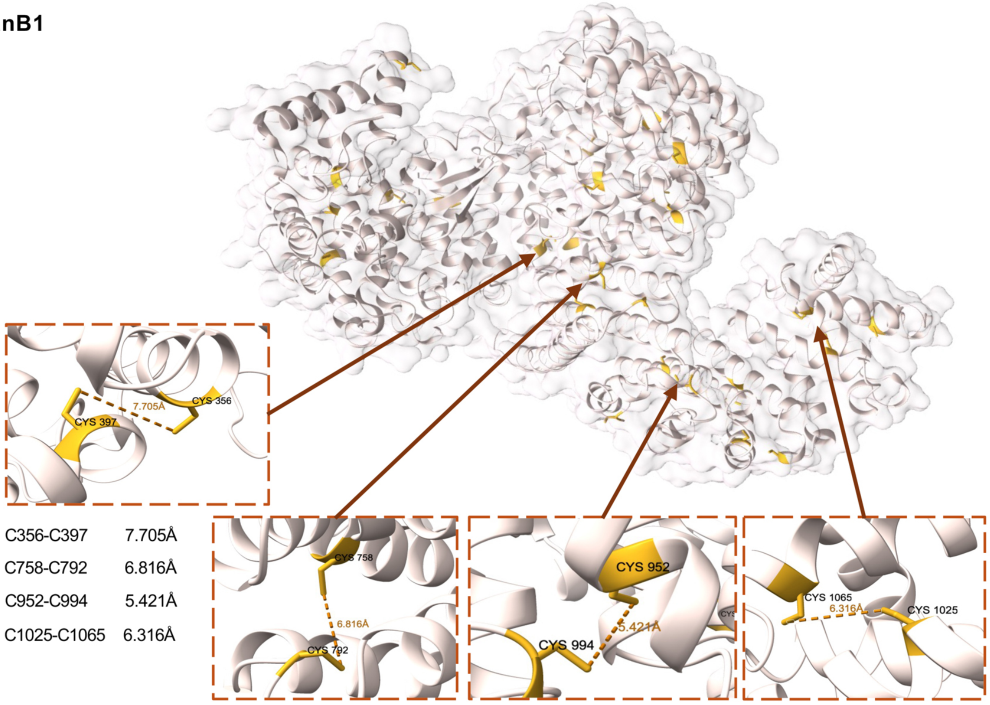

Journal of Cell Science • Supplementary information

Ipo5

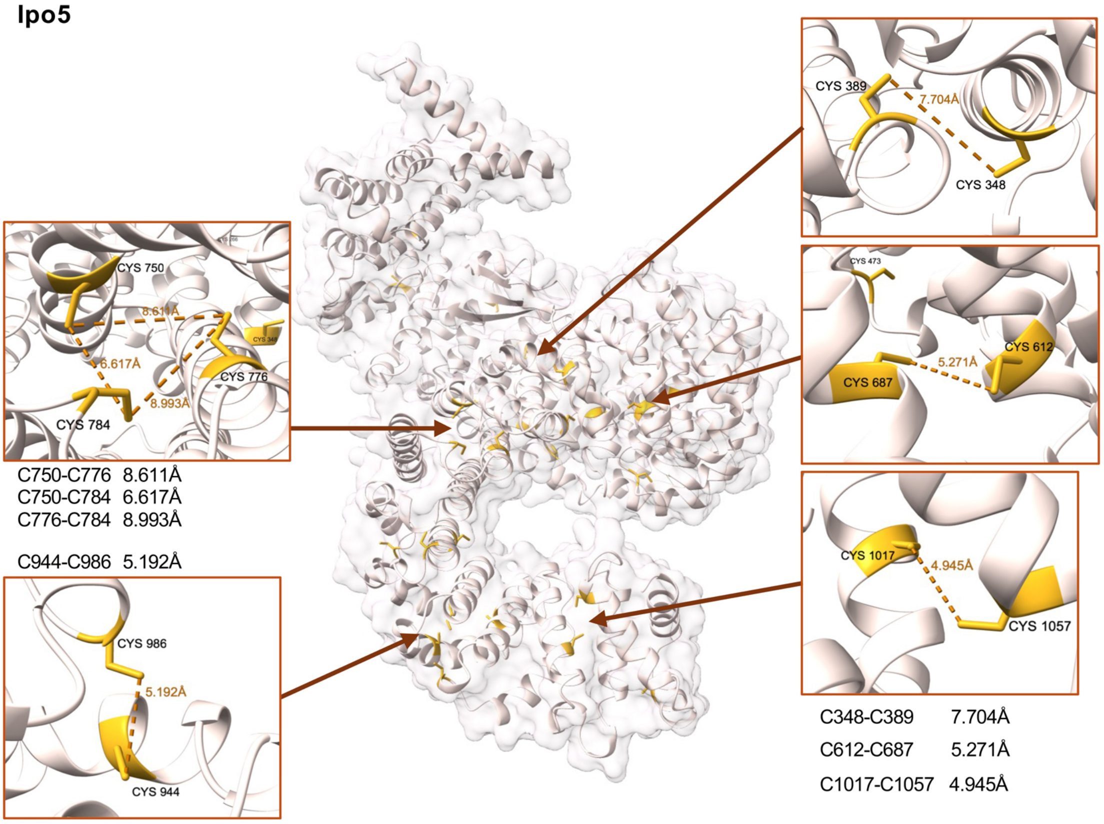
CYS 750
8.611Å
6.617Å
CYS 776
8.993Å
CYS 784
C750-C776 8.611Å
C750-C784 6.617Å
C776-C784 8.993Å
C944-C986 5.192Å
CYS 986
5.192Å
CYS 944
CYS 389
7.704Å
CYS 348
CYS 473
CYS 612
5.271Å
CYS 687
CYS 1017
4.945Å
CYS 1057
C348-C389 7.704Å
C612-C687 5.271Å
C1017-C1057 4.945Å

Nup188

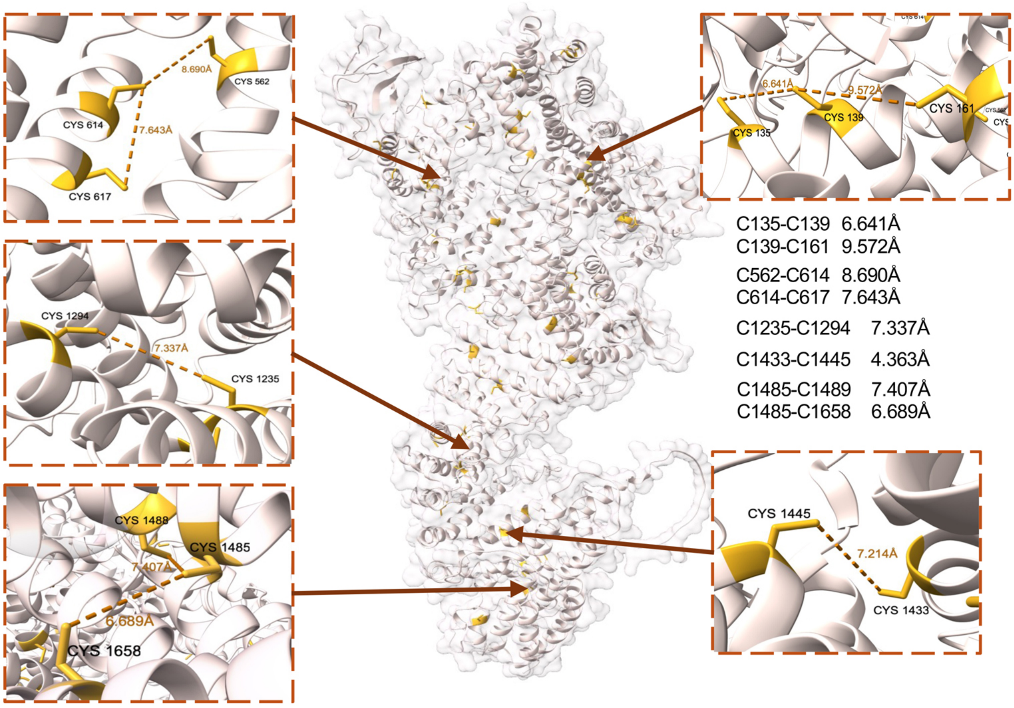
8.690Å
CYS 562
CYS 614
7.643Å
CYS 617
CYS 1294
7.337Å
CYS 1235
CYS 1488
CYS 1485
7.407Å
6.689Å
CYS 1658
6.641Å
9.572Å
CYS 135
CYS 139
CYS 161
C135-C139 6.641Å
C139-C161 9.572Å
C562-C614 8.690Å
C614-C617 7.643Å
C1235-C1294 7.337Å
C1433-C1445 4.363Å
C1485-C1489 7.407Å
C1485-C1658 6.689Å
CYS 1445
7.214Å
CYS 1433

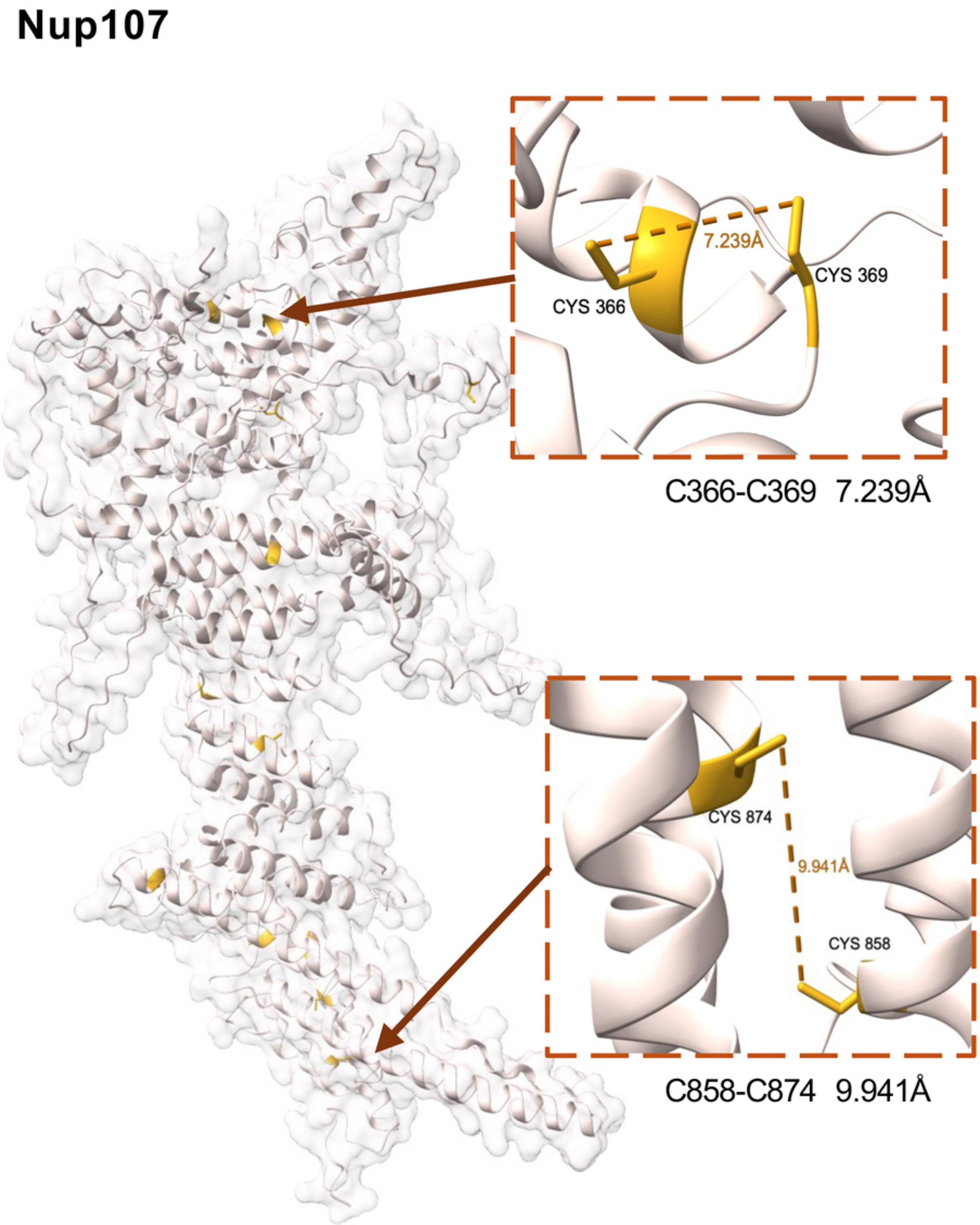

Fig. S6. AlphaFold structure predictions and cysteine mapping for human orthologues of yeast Kap95 (KPNB1), Kap121 (IPO5 and RANBP6), Nup84 (NUP107) and Nup188 (NUP188). Distances between pairs of adjacent or proximal cysteines are indicated. The structure predictions are based on experimental crystal structure data for all proteins.

Journal of Cell Science • Supplementary information

A

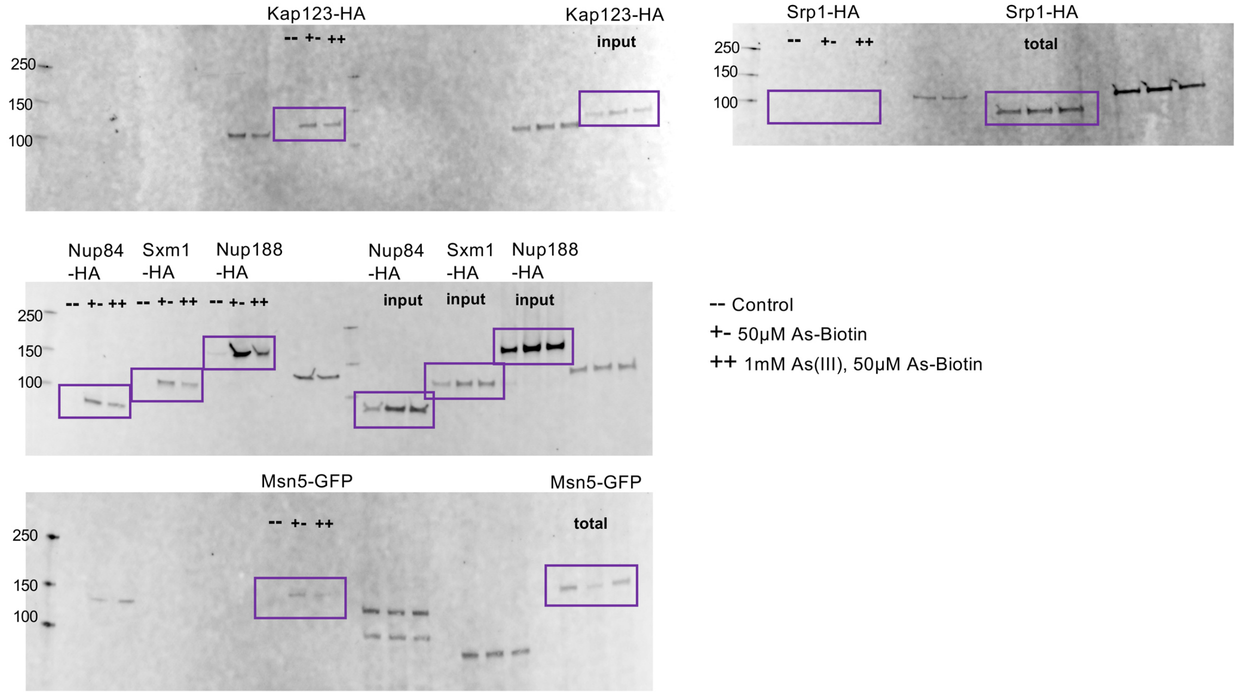
Kap123-HA
-- +- ++
Kap123-HA
input
Srp1-HA
-- +- ++
Srp1-HA
total
250
150
100
Nup84 -HA
Sxm1 -HA
Nup188 -HA
-- +- ++ -- +- ++ -- +- ++
Nup84 -HA
Sxm1 -HA
Nup188 -HA
input
input
input
-- Control
+- 50μM As-Biotin
++ 1mM As(III), 50μM As-Biotin
Msn5-GFP
-- +- ++
Msn5-GFP
total

B

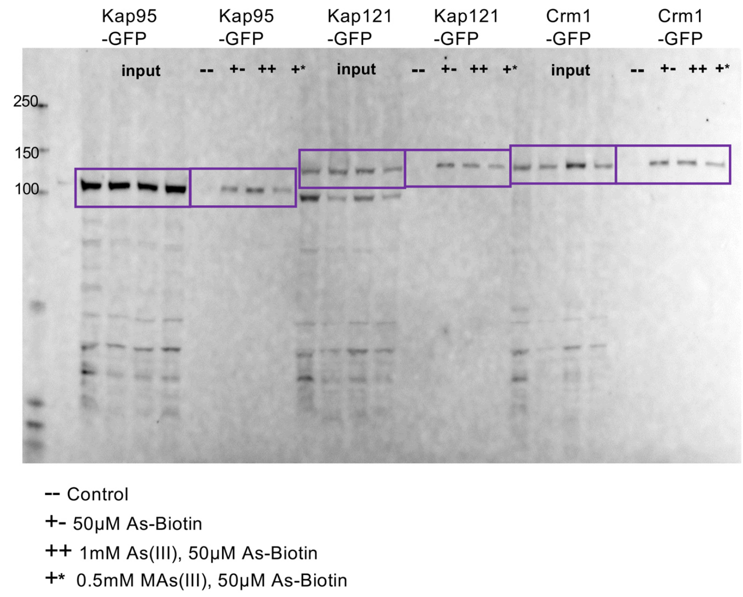
Kap95 -GFP
Kap95 -GFP
Kap121 -GFP
Kap121 -GFP
Crm1 -GFP
Crm1 -GFP
input
-- +- ++ +*
input
-- +- ++ +*
input
-- +- ++ +*
250
150
100
-- Control
+- 50μM As-Biotin
++ 1mM As(III), 50μM As-Biotin
+* 0.5mM MAs(III), 50μM As-Biotin

C

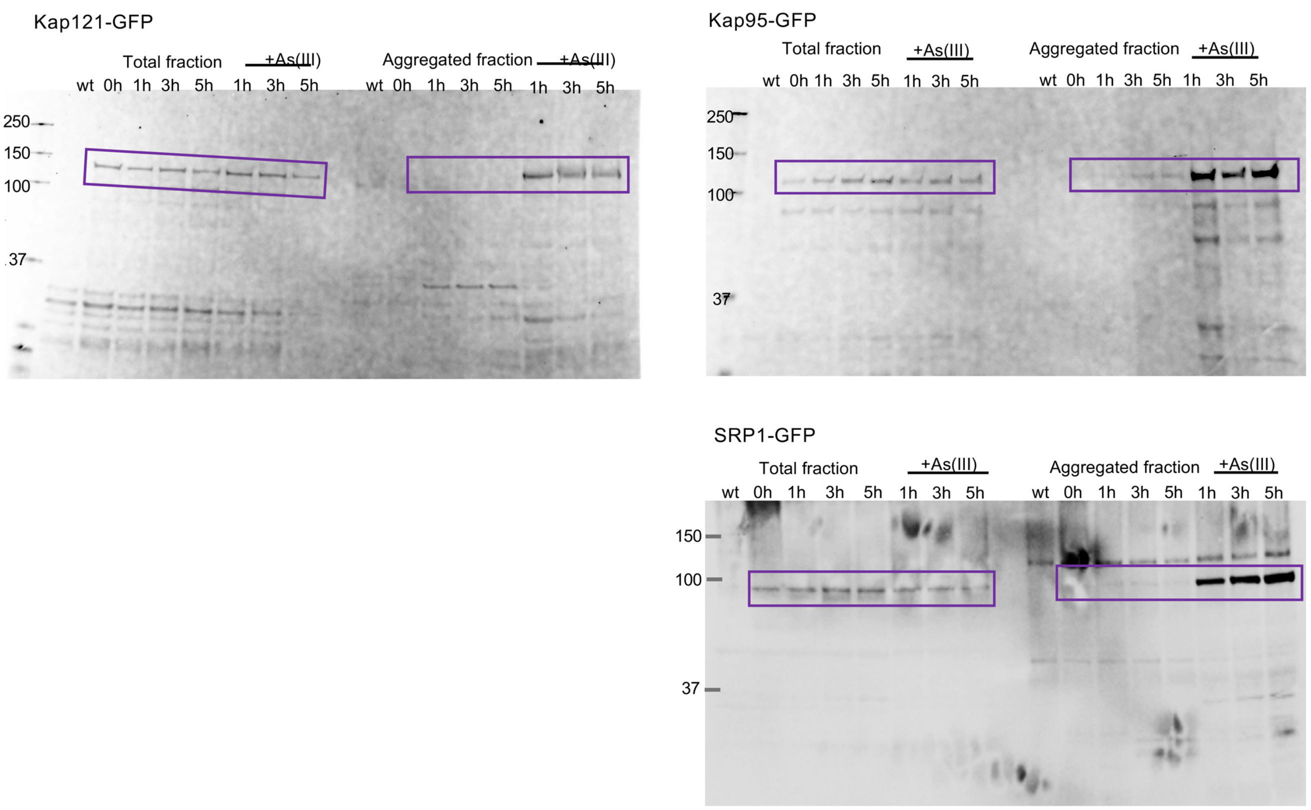

Fig. S7. Blot transparency. Unprocessed Westen blot images presented in this study corresponding to Fig. 2B (in A), Fig. 2C (in B) and Fig 3D (in C).C)

Table S1. All proteins detected by LC-MS/MS.

Available for download at

https://journals.biologists.com/jcs/article-lookup/doi/10.1242/jcs.263889#supplementary-data

Table S2. List of 174 candidate arsenic-binding proteins.

Available for download at

https://journals.biologists.com/jcs/article-lookup/doi/10.1242/jcs.263889#supplementary-data

Table S3. List of strains and plasmids used.

Available for download at

https://journals.biologists.com/jcs/article-lookup/doi/10.1242/jcs.263889#supplementary-data

Table S4. Datasets used for comparisons.

Available for download at

https://journals.biologists.com/jcs/article-lookup/doi/10.1242/jcs.263889#supplementary-data
